# Dual-responsive degradable core–shell nanogels with tuneable aggregation behaviour†

**DOI:** 10.1039/d1ra07093b

**Published:** 2022-01-13

**Authors:** Dominic M. Gray, Adam R. Town, Edyta Niezabitowska, Steve P. Rannard, Tom O. McDonald

**Affiliations:** Department of Chemistry, University of Liverpool Crown Street L69 7ZD UK Thomas.Mcdonald@liverpool.ac.uk Dominic.Gray@liverpool.ac.uk; Materials Innovation Factory, University of Liverpool Crown Street L69 7ZD UK

## Abstract

We report the synthesis of core–shell nanogels by sequential addition of thermoresponsive monomers; *N*-isopropylacrylamide (NIPAM) and *N*-isopropylmethacrylamide (NIPMAM). The aggregation behaviour of aqueous dispersions of these particles in the presence of salt can be tuned by varying the monomer ratio. The inclusion of degradable cross-linker bis(acryloyl)cystamine (BAC) allows the nanogels to degrade in the presence of reducing agent, with nanogels composed of a copolymer of the two monomers not showing the same high levels of degradation as the comparable core–shell particles. These levels of degradation were also seen with physiologically relevant reducing agent concentration at pH 7. Therefore, it is hoped that the aggregation of these nanogels will have applications in nanomedicine and beyond.

## Abstract 2

Core–shell nanogels with a poly(*N*-isopropylmethacrylamide) core and poly(*N*-isopropylacrylamide) shell display tuneable thermoresponsive behaviour and high degradability.

## Graphical abstract

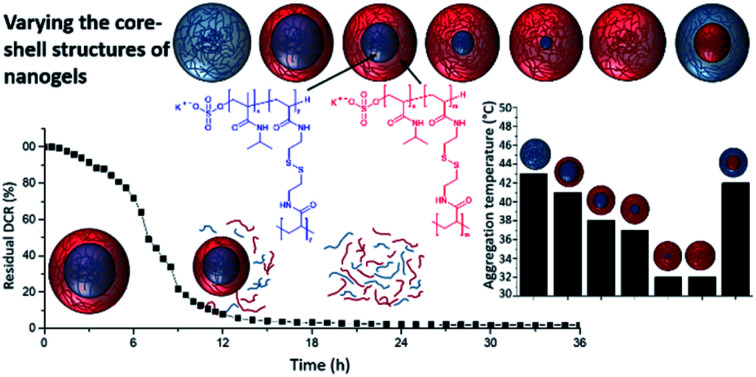

## Introduction

A nanogel is a nanoscale (1–1000 nm) particle consisting of a cross-linked polymer network, which has the ability to be swollen by a good solvent, usually water.^1^ These nanogels can display responsive behaviour to environmental stimuli such as temperature, pH and ionic strength.^2^ Thermoresponsive nanogels can be prepared from such polymers as poly(*N*-isopropylacrylamide) (PNIPAM),^3^ poly(*N*-vinylcaprolactam),^4,5^ and poly(oligo(ethylene glycol) methacrylate).^6^ PNIPAM nanogels have a volume phase transition temperature (VPTT) in water at approximately 33 °C,^7,8^ making these materials interesting for healthcare applications. Above the VPTT, the polymer–polymer interactions within the nanogel become favourable compared to polymer–water interactions, ejecting water and decreasing the hydrodynamic diameter of the particles. Such a deswelling event can also influence a range of other nanoparticle properties including charge density, light scattering and hydrophobicity. By careful design of the nanogels the behaviour associated with the VPTT can be used to trigger changes in the colloidal stability of the particles.^9^ Below the VPTT, nanogels are sterically stabilised by polymer chain ends extending out from the particle into solution.^10^ This steric stabilisation is lost above the VPTT. Charges present on the nanogels, typically from the use of a charged initiator, can provide electrostatic repulsion between the nanogels and provide colloidal stabilisation of the particles above the VPTT.^10^ This electrostatic repulsion may be screened out by ions present in solution, which can result in nanogel aggregation. Therefore, these nanogels are responsive to both temperature and ionic strength, making them dual responsive. The dual-responsive nature allows these particles to aggregate under specific conditions and are otherwise dispersed until these conditions are reached. As a result, these aggregates are of interest in applications such as *in situ* forming systems,^11–15^ colloidal catalysis,^16,17^ and pore blocking.^18,19^

A potential limitation to the use of PNIPAM nanogels for biomedical applications is their persistent nature; the polymer shows very limited degradability and thus could potentially accumulate within the body. It is hoped that degradable nanogels could be used in biomedical applications, such as *in situ* forming systems, and the depot formed would degrade and be cleared by the body. This would negate the need for removal of any remaining materials by a medical professional after the drug release is complete. Nanogel degradation has been shown to be possible *via* the incorporation of cross-linkers that can undergo cleavage and removing the cross-linking within the nanogel network. A range of different degradable cross-linkers have been utilised in nanogel synthesis which can degrade in response to various conditions, such as pH and the presence of enzymes or reducing agents.^20–23^ Examples of these include; 2-bis[2,2′-di(*N*-vinylformamido)ethoxy]propane,^24,25^ dextran methacrylate,^26,27^ and *N*,*N*′-bis(acryloyl)cystamine (BAC).^28–31^ The latter of these is perhaps the most widely used biodegradable cross-linking agent employed in numerous PNIPAM nanogels. The disulfide bonds with BAC can undergo cleavage by reduction under physiologically relevant reducing conditions.^31^ Within the body, the main disulfide bond reducing agent is glutathione (GSH), which is found at a concentration of 2–20 μM in the extracellular environment, and 0.5–10 mM in the intracellular environment.^32^ The presence of these reducing agents could potentially allow BAC cross-linked nanogels to be slowly degraded in an extracellular environment, generating low molecular weight polymers, which can be eliminated from the body. In order for nanogel degradation products to be removed through renal excretion, it has been shown that these products should be less than 40 kDa in molecular weight to be rapidly cleared.^33^ Note, this was shown for 2-hydroxypropyl methacrylamide and may be polymer dependant. For *in vitro* experiments, dithiothreitol (DTT) is often used to trigger the reduction of disulfide bonds to mimic the role of GSH in the body.^28,30,34^ In order to assess the degradation of nanogels a range of techniques have been employed, these include asymmetric flow field flow fractionation,^18,28^ dynamic light scattering,^20,35^ atomic force microscopy,^36,37^ scanning electron microscopy,^31,38^ visual turbidity,^39,40^ and indirectly through enhanced drug or dye release.^29,41^ Many PNIPAM nanogels cross-linked with the degradable cross-linking agents have tended to display incomplete degradation. For example, (1,2-dihydroxylethylene)bisacrylamide cross-linked particles have previously been reported to give incomplete degradation.^18^ It was hypothesised that the hydrogen atom of the tertiary carbon of the polymer backbone of PNIPAM and the tertiary carbon proton of the isopropyl group can be abstracted, in a chain transfer reaction, to form permanent cross-links.^36,42–44^ Further studies found that replacing the NIPAM monomer with *N*-isopropylmethacrylamide (NIPMAM) (identical in structure except that NIPMAM has a methyl group in place of the hydrogen atom present in NIPAM on the tertiary carbon of the polymer backbone^45^) could produce fully degradable nanogels.^18^ In other work, Gaulding *et al.*, synthesised PNIPMAM nanogels with the degradable cross-linking agent BAC.^28^ They observed incomplete degradability, which was attributed to the elevated temperature of synthesis (80 °C) allowing the disulfide bond of the BAC cross-linking agent to form non-degradable thioether cross-links. However, fully degradable nanogels were synthesised by conducting low temperature redox-initiated polymerisation (50 °C) but required the addition of accelerant *N*,*N*,*N*′,*N*′-tetramethylethylenediamine (TEMED).^28^ Without the use of TEMED, self-cross-linking of PNIPAM particles can be achieved at temperatures as low as 25 °C, depending on reaction conditions.^44^ It is apparent that synthesising degradable PNIPAM nanogels can often require synthesis conditions at limit the formation of non-degradable cross-links.

As a thermoresponsive material, the specific VPTT of the nanogels is critical to their behaviour. Some of the modifications used to achieve degradable behaviour directly impact the VPTT. For example, PNIPMAM nanogels exhibit a VPTT between 43 and 46 °C.^46^ As this is above body temperature, nanogels composed purely of PNIPMAM will not exhibit any response to the trigger of a temperature increase to body temperature. Studies on non-degradable nanogels have shown it is possible to tune the VPTT on nanogels by combining NIPMAM and NIPAM monomers and varying the monomer feed ratio/polymer composition.^47^ Nanogels synthesised with a 60 : 40 mol% NIPAM : NIPMAM monomer ratio, had a VPTT of 33.7 °C. Weise *et al.* similarly showed that a 50 : 50 monomer molar ratio gave nanogels with a VPTT of 38 °C.^48^ Another approach that has been reported is to synthesise nanogels with a PNIPAM core and PNIPMAM shell structure. The synthesis of core–shell particles, consisting of a chemically distinct polymer shell to the core, allows for potentially superior particle properties compared to the individual components.^49^ Particles composed of a core–shell structure using 70 : 30 PNIPAM : PNIPMAM monomer ratio had a higher aggregation temperature (*T*_agg_ = 40 °C) than the copolymer nanogel composed of the same mol% of each monomer (36 °C).^48^ In such core–shell structures, the *T*_agg_ will be heavily dependent on the VPTT of the shell rather than the core. All prior studies on PNIPAM–PNIPMAM copolymers and core–shell nanogels have been non-degradable, and as such, there is the potential to use core–shell structures in nanogels that can combine switchable changes in colloidal stability combined with degradability by using a PNIPAM shell and a PNIPMAM core.

**Fig. 1:**
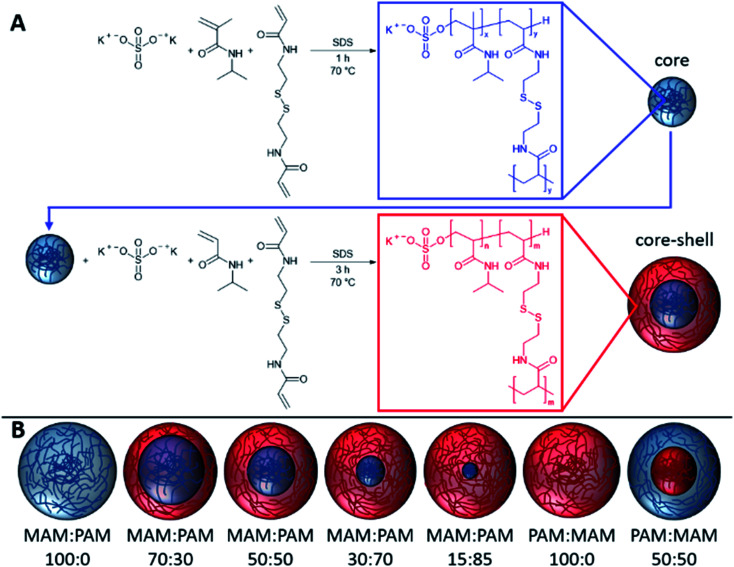
The synthesis of core–shell nanogels consisting of MAM (*N*-isopropylmethacrylamide) and PAM (*N*-isopropylacrylamide) and using *N*,*N*′-bis(acryloyl)cystamine (BAC) as the cross-linker. Different core–shell nanogels were synthesised by varying the ratio of core and shell monomers.

In this study, we prepare highly degradable nanogels with a VPTT below body temperature. To achieve this, the effect of monomer ratio of NIPAM and NIPMAM in the formation of degradable core–shell nanogels was investigated (Fig. 1). These nanogels were characterised in terms of their thermoresponsive and degradation behaviour. For future use in the intended biomedical applications, the nanogels should aggregate below body temperature (37 °C) and degrade to low molecular weight, or <20 nm in diameter, species so the degradation products can be removed by the body. For the naming convention of all samples, the core forming monomer is listed first, either NIPAM (PAM) or NIPMAM (MAM), with the secondary monomer used to synthesise the shell. This is followed by the molar ratio of the monomer in the core and shell, respectively. These parameters were determined by dynamic light scattering (DLS) and their degradation inferred by comparisons of residual derived count rates before and after degradation. The degradation behaviour of selected nanogels was then investigated under physiologically relevant conditions to show high levels of degradation of the nanogels.

## Experimental

### Materials

*N*-Isopropylacrylamide (NIPAM, ≥99%), *N*-isopropylmethacrylamide (NIPMAM, 97%), *N*,*N*′-bis(acryloyl)cystamine (BAC, 98%), potassium persulfate (KPS, ≥99%), anhydrous sodium hydroxide pellets (NaOH, analysis grade), sodium dodecyl sulphate (SDS, ≥99%), 1,4-dithiothreitol (DTT, >97%), deuterium oxide (99.9% atom D, containing 1 wt% 3-(trimethylsilyl)-1-propanesulfonic acid, sodium salt) were purchased from Sigma-Aldrich Company Ltd, Gillingham (Dorset) UK, a subsidiary of Merck KGaA, Darmstadt, Germany and used as received. Phosphate buffered saline tablets (Bioreagent), purchased from Fischer Scientific UK, Loughborough, UK, a part of Thermo Fisher Scientific. Type I distilled water obtained from a water purification system with a resistivity of >18 MΩ cm^−1^ (PURELAB option R, Veolia). Spectra/por 2 (MWCO = 12–14 kDa) dialysis tubing was purchased from Spectrum Europe B.V., Breda, The Netherlands.

### Nanogel synthesis

The PNIPAM nanogels were synthesised by dispersion polymerisation. The composition used in the synthesis of each nanogel can be found in Table 1. The nanogels discussed in detail in the particle degradation section of this publication were also synthesised on a ×2.5 smaller scale than the scale given in Table 1. This was found not to affect the results and so data has been combined so values do not change throughout (ESI Table 1†). For the synthesis of the core, the NIPAM or NIPMAM monomer, BAC cross-linking agent and SDS surfactant were dissolved in distilled water in a 250 mL two-neck round bottom flask equipped with a stirrer bar and reflux condenser. This was then sealed and nitrogen was bubbled through the aqueous solution for 1 h whilst stirring (400 rpm) to remove dissolved oxygen. The solution was then heated to 70 °C. Separately KPS initiator was dissolved in distilled water (9.38 mg mL^−1^) and purged with nitrogen for 1 h before being transferred to the flask containing the monomers. The reaction was maintained under a nitrogen atmosphere for 1 h at 70 °C before further addition of the shell monomer, cross-linking agent and SDS which were separately sealed and degassed with nitrogen for 1 h whilst stirring (400 rpm), and further KPS initiator solution. After a further 3 h at 70 °C the solution was cooled down to room temperature. Where only a core was synthesised, the reaction was simply conducted for 4 h to give the same total reaction time. To remove unreacted impurities, the nanogel suspension was dialysed for 5 days using regenerated cellulose dialysis tubing (12–14 kDa MWCO, Spectrum Labs), replacing the distilled water twice daily. After dialysis, samples were lyophilised using a Virtis benchtop K under vacuum for 72 h and redispersed with shaking at the required concentrations.

**Table 1:** Reagent breakdown for each synthesised nanogel

Sample	Monomer^a^		BAC^b^		KPS^c^		SDS		Water^d^ (g)	
	NIPMAM	NIPAM	Core	Shell	Core	Shell	Core	Shell	Core	Shell
MAM:PAM 100:0	34.7 mmol	—	451.8 mg	—	187.6 mg	—	80 mg	—	140	—
	4.414 g		1.735 mmol		0.694 mmol		0.277 mmol			
MAM:PAM 70:30	3.087 g	1.180 g	316.28 mg	135.54 mg	131.4 mg	56.28 mg	56 mg	24 mg	98	42
	24.29 mmol	10.41 mmol	1.215 mmol	0.521 mmol	0.486 mmol	0.208 mmol	0.194 mmol	0.083 mmol		
MAM:PAM 50:50	2.207 g	1.963 g	225.9 mg	225.9 mg	93.8 mg	93.8 mg	40 mg	40 mg	70	70
	17.35 mmol	17.35 mmol	0.868 mmol	0.868 mmol	0.347 mmol	0.347 mmol	0.139 mmol	0.139 mmol		
MAM:PAM 30:70	1.324 g	2.749 g	135.54 mg	316.26 mg	56.2 mg	131.38 mg	24 mg	56 mg	42	98
	10.41 mmol	24.29 mmol	, 0.521 mmol	1.215 mmol	0.208 mmol	0.486 mmol	0.083 mmol	0.194 mmol		
MAM:PAM 15:85	0.6624 g	3.340 g	67.77 mg	384.0 mg	28.14 mg	159.46 mg	12 mg	68 mg	21	119
	5.25 mmol	29.50 mmol	0.260 mmol	1.475 mmol	0.104 mmol	0.590 mmol	0.042 mmol	0.236 mmol		
PAM:MAM 100:0	—	3.924 g	451.8 mg	—	187.6 mg	—	80 mg	—	—	140
		34.7 mmol	1.735 mmol		0.694 mmol		0.277 mmol			
PAM:MAM 50:50	2.207 g	1.963 g	225.9 mg	225.9 mg	93.8 mg	93.8 mg	40 mg	40 mg	70	70
	17.35 mmol	17.35 mmol	0.868 mmol	0.868 mmol	0.347 mmol	0.347 mmol	0.139 mmol	0.139 mmol		

a 34.7 mmol of total NIPAM and/or NIPMAM used in synthesis.

b 5% of monomer moles.

c 2% of monomer moles as 9.38 mg mL^−1^ aqueous solution.

d 160 mL total reaction volume including 20 mL aqueous KPS solution.

### Analysis

#### Nuclear magnetic resonance (NMR)

All ^1^H NMR was carried out on a Bruker Avance III 400 MHz NMR. All spectra were obtained using deuterium oxide containing 1 wt% 3-(trimethylsilyl)-1-propanesulfonic acid, sodium salt as reference standard. Water suppression was achieved using a Noesy presat pulse program with a 10 mS mixing time.

#### DLS and electrophoretic mobility measurements

To characterise the nanogels after synthesis dynamic light scattering (DLS) measurements were performed at 25 °C with a 1 mg mL^−1^ nanogel dispersion using an equilibration time of 240 seconds, unless otherwise stated, with a Malvern Zetasizer Nano ZS (running Malvern Zetasizer software V7.12) (Malvern Instruments, Malvern, UK) with 633 nm He–Ne laser and the detector positioned at 173°. Material refractive index was set as 1.520. Measurement duration (run number and duration), position and attenuator selection were all selected automatically. 1 cm path length disposable polystyrene cuvettes were used for measurements. Measurements were repeated in triplicate to give a mean *D*_h_ and polydispersity index (PDI) value.

Particle degradation is inferred from derived count rate obtained from DLS measurements carried out as stated here, unless specified in the main text. Samples were analysed at 1 mg mL^−1^ pH 10 aqueous dispersions, pH adjustments were made using 1 M NaOH solution. Degradation was achieved using 150 mM DTT concentration to degrade particles rapidly. Measurements were conducted at the same measurement position (4.65 mm, centre of the cuvette) with automatic attenuator selection and measurement duration. Measurements were obtained using the same instrument described previously, at 173° and 240 seconds equilibration time. Nanogel degradations carried out under physiologically relevant conditions were completed as described for the degradations above but using 10 mM DTT concentration at pH 7. DLS measurements were obtained at 0.5 h intervals for the first 12 h after the addition of DTT and 1 h time intervals after.

Volume phase transition temperature (VPTT) in de-ionised water and particle aggregation temperatures in phosphate buffer saline (PBS) solution (0.01 M phosphate bugger, 0.0027 M potassium chloride and 0.137 M sodium chloride, pH 7.4 at 25 °C) were obtained using the same base DLS parameters as previously mentioned. The temperature of the sample was equilibrated at 15 °C for 12 minutes and each increment in temperature was equilibrated for 4 minutes up to 55 °C. The temperature was raised in 0.5 °C increments around the expected VPTT values of the nanogel, and 1 °C per measurement above and below these values.

Zeta potential measurements were also conducted using a Malvern Zetasizer Nano ZS. Samples were analysed in DTS1070 Zetasizer Nano series disposable folded capillary cells. Nanogels were dispersed in a 1 mg mL^−1^, 1 mM aqueous solution of NaCl as background electrolyte and analysed using the Smoluchowski model at 25 °C. Measurements consisted of between 10 and 50 runs and measured in triplicate with an average result taken with automatic attenuation and voltage selections.

## Results and discussion

### Synthesis of core–shell nanogels

Nanogels were synthesised by the polymerisation of the core monomers with BAC, followed by shell monomers with BAC to provide core–shell nanogels. Seven nanogel compositions were prepared in total. Four were synthesised by decreasing the amount of PNIPMAM used as the core while increasing the amount of NIPAM used to synthesise the shell. Two more nanogels were prepared by forming only a core using either monomer. A NIPMAM core was selected in order to provide high degradability of the nanogels, while the NIPAM shell was used to obtain the appropriate VPTT behaviour. As the shell polymer has more of an effect on the VPTT than the core.^48^ A control sample of a PNIPAM core and PNIPMAM shell was also prepared in order to determine the effect that the shell polymer had on the thermoresponsive behaviour. The resulting nanogels were characterised by DLS to obtain a mean diameter, PDI and VPTT value for each nanogel composition, see Table 2. All the of resulting nanogels were found to have hydrodynamic diameters between 96–208 nm and very low polydispersity index (PDI) (0.02–0.04), as expected for successful dispersion polymerisations. There were no clear trends observed in the relationship between hydrodynamic diameter and the composition of the nanogels.

**Table 2:** Composition and properties of degradable core–shell nanogels using the monomers NIPAM and NIPMAM

Sample	NIPMAM^a^ mol%	NIPAM^a^ mol%	Hydrodynamic diameter^b^ (nm) ± St Dev.^c^	PDI	Zeta potential^d^ (mV)	Swelling ratio^e^	*T*_agg_ (°C)
MAM:PAM 100:0	100	0	143 ± 1.2	0.02	−18	1.35	43
MAM:PAM 70:30	70	30	205 ± 2.0	0.02	−26	1.40	41
MAM:PAM 50:50	50	50	153 ± 3.3	0.01	−29	1.28	38
MAM:PAM 30:70	30	70	171 ± 1.6	0.02	−32	1.28	37
MAM:PAM 15:85	15	85	173 ± 0.8	0.04	−25	1.18	32
PAM:MAM 100:0	0	100	82 ± 0.8	0.02	−30	1.24	32
PAM:MAM 50:50	50	50	213 ± 5.4	0.03	−19	1.50	42

a mol% based on total moles (34.7 mmol) of NIPMAM and NIPAM, excludes moles of cross-linker and initiator used.

b Hydrodynamic diameter of an aqueous dispersion at 25 °C and 1 mg mL^−1^ using DLS with the mean value of triplicate measurements. Particle size distribution graphs shown in ESI Fig. 1

c Standard deviation from running of sample in triplicate.

d Nanogels were analysed as a 1 mg mL^−1^ aqueous dispersion using 1 mM NaCl background electrolyte concentration at 25 °C.

e Swelling ratio calculated using *D*_h_ (hydrodynamic diameter). *D*_h_ (15 °C)/*D*_h_ (55 °C).

**Fig. 2:**
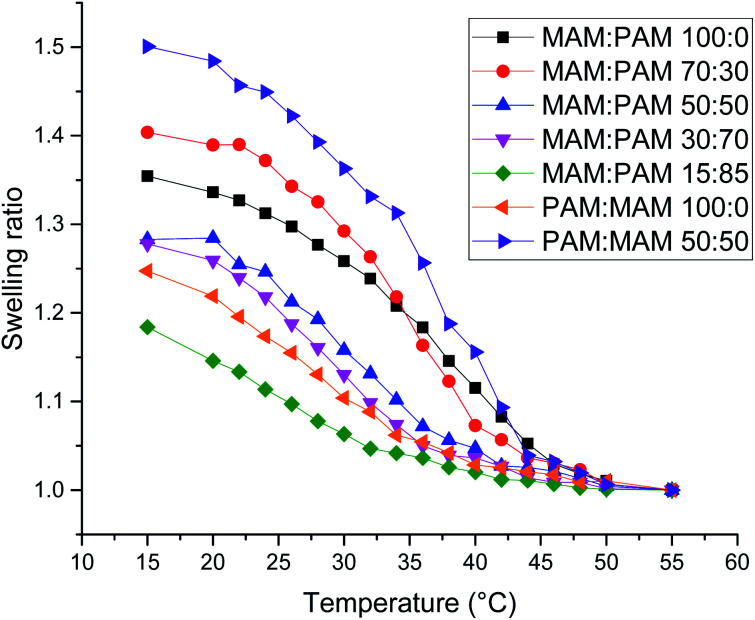
Swelling ratio against temperature of various core–shell nanogels. Swelling ratio was calculated using *D*_h_ values of the nanogels at that temperature compared to the value at 55 °C.

**Fig. 3:**
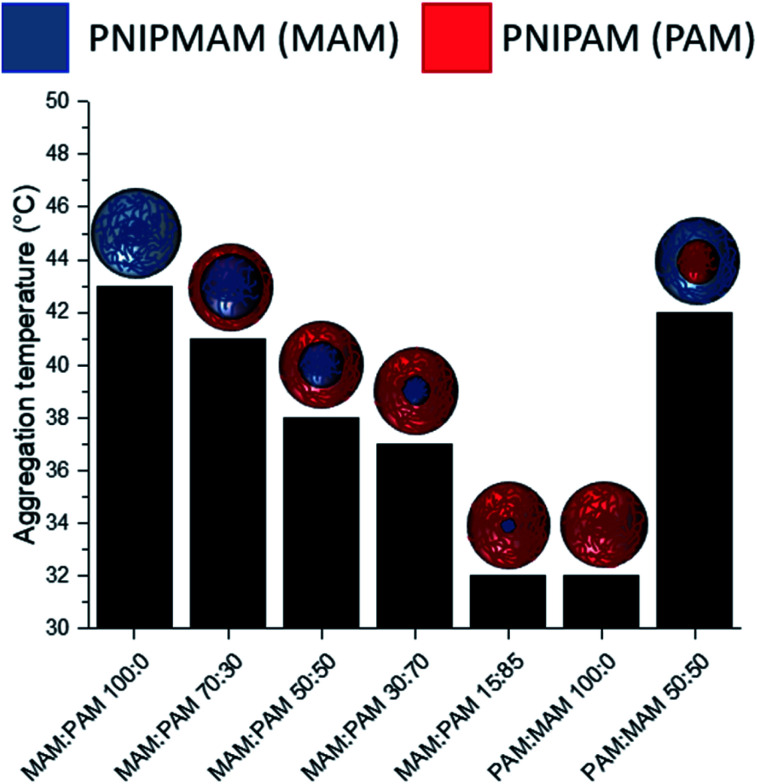
The effect of nanogel structure and composition with varying monomer ratio of core and shell additions of NIPAM and NIPMAM on the *T*_agg_. MAM:PAM denoting a NIPMAM core and NIPAM shell and reversed for PAM:PAM nanogels.

There was a general trend that the swelling ratio (a ratio of a swollen hydrodynamic diameter (*D*_h_) (15 °C) to the deswollen *D*_h_ (55 °C) increased with PNIPAM content (Table 2)). Šťastná *et al.* reported similar findings when investigating PNIPAM/PNIPMAM interpenetrating polymer networks.^50^ This behaviour was likely due to the more hydrophilic nature of PNIPAM compared to PNIPMAM. The thermoresponsive behaviour of different nanogels as seen by a decreasing swelling ratio with increasing temperature for each nanogel batch, can be found in Fig. 2. The aggregation temperature (*T*_agg_) in PBS was then investigated. It was found to decrease with increasing amounts of NIPAM in the monomer ratio (Fig. 3). This aggregation behaviour is due to the combination of electrostatic and steric colloidal stabilisation displayed by the nanogels. Below the *T*_agg_ the nanogels have both steric and electrostatic repulsion, the nanogels had zeta potential values of −18 to −32 mV due to the persulfate polymer chain-end groups present on the surface of the particles. This charge stabilisation was also enough to stabilise the particles above the VPTT over short timescales as shown by *D*_h_ and PDI values of the particles by DLS. In the presence of PBS, the salts screen the electrostatic repulsion arising from the anionic initiator fragments, leaving only the steric repulsion from the solvated polymer chains on the surface of the nanogels. When the temperature is increased above the VPTT of the shell polymer, the surface chains become desolvated and no longer provide steric repulsion. Without any repulsive interactions between the nanogels the particles aggregate. As seen in a previous publication, the shell monomer has a greater effect on the *T*_agg_ of the particles.^48^ PNIPAM has a lower VPTT and therefore the properties of the shell increasingly dominate the impact on colloidal stability as the more hydrophilic PNIPAM shell becomes thicker. The effect of the shell polymer can be seen if we compare MAM:PAM 50:50 to PAM:MAM 50:50, as the *T*_agg_ was 4° higher when the higher LCST polymer PNIPMAM was used as the shell rather than the core. These differences in the thermoresponsive behaviours of the different nanogels provide evidence towards the existence of core–shell structure of the nanogels; the VPTT was controlled by the monomer added second during in the polymerisation resulting in the formation of the nanogel shell.

MAM:PAM 15:85 was the only sample containing PNIPMAM with a *T*_agg_ < 37 °C, a behaviour necessary for these nanogels to aggregate in physiological conditions. As a result, we will examine these particles and their degradability in more detail.

### Particle degradation

The degradation of nanogels can be investigated through dynamic light scattering, allowing degradation to be monitored *in situ*. Chen *et al.* and Leber *et al.* showed that the count rate of a nanogel sample drops as the nanogel degrades.^20,35^ Assuming that particles do not reduce in concentration through the processes such as aggregation or sedimentation, then a drop in derived count rate (DCR) over time can be attributed to a change in refractive index of the particles, a reduction in mean particle diameter and/or a reduction in the particle concentration due to particle degradation. In order to initially test the degradability of these nanogels, pH 10 and 150 mM concentration of dithiothreitol (DTT) was used at room temperature. These degradation conditions were selected in order to achieve rapid degradation to accelerate testing of the samples (photographic evidence can be seen in ESI Fig. 2†). The increased pH was also used to increase the rate of degradation as more DTT exists in the active thiolate form capable of degrading the disulfide cross-linked (DTT thiol p*K*_a_ values are 9.2 and 10.1).^51^

In order to investigate the effect of composition and core–shell structure of the nanogels MAM:PAM 100:0 and PAM:MAM 100:0 were subjected to degradation conditions as standards along with the chosen MAM:PAM 15:85 sample. All samples that underwent degradation studies displayed no visual evidence of aggregation or sedimentation and therefore changes in the DCR can be closely linked to particle degradation. For particles consisting of solely PNIPAM (PAM:MAM 100:0) the residual DCR was 26.7% after being exposed to degradation conditions, Table 3. Particles consisting of only PNIPAM were less likely to fully degrade due to the formation of permanent cross-links within these particles.^36,42–44^ However, particles consisting of only PNIPMAM (MAM:PAM 100:0) degraded to 2.5% of the original DCR. This level of degradation was much more extensive compared to that reported by Gaulding *et al.* however, this difference might be attributed to our lower synthesis temperature 70 °C (rather than at 80 °C used by Gaulding *et al.*).^28^

**Table 3:** Table of dispersion analyses before and after degradation at pH 10 and 150 mM DTT concentration. Conversion obtained by ^1^H NMR included also

Sample	Residual derived count rate	*D*_h_ before/nm (PDI)	*D*_h_ after/nm (PDI)	Conversion by ^1^H NMR
PAM:MAM 100:0	25%	90 (0.02)	125 (0.03)	PAM 35%^a^
MAM:PAM 100:0	2.5%	143 (0.02)	163 (0.34)	MAM 56%, BAC 85%
MAM:PAM 15:85	5.6%	173 (0.04)	173 (0.23)	1 h = MAM 55% and BAC 85%
				Final = MAM 69%, PAM 19%^a^
MAM:PAM 15:85 not core–shell	24%	105 (0.02)	186 (0.02)	MAM 46%
				PAM 30%^a^

a BAC conversion could not be estimated due to the overlap of ^1^H NMR peaks with NIPAM.

**Fig. 4:**
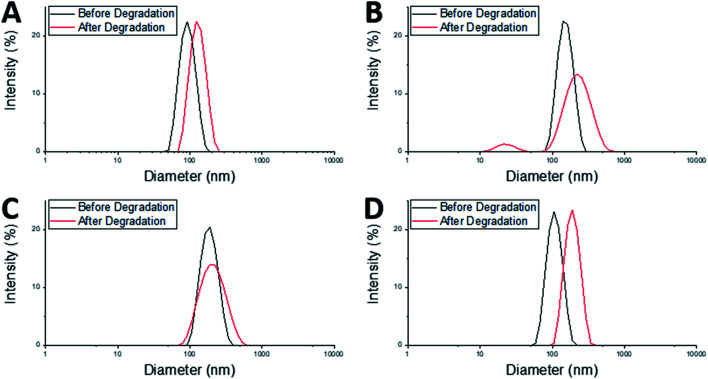
Particle size distributions of nanogels before degradation at 1 mg mL^−1^ at 25 °C pH 7 (black), and after degradation at 1 mg mL^−1^ concentration, 25 °C, 150 mM DTT concentration at pH 10 (red) of particles; (A) PAM:MAM 100:0, (B) MAM:PAM 100:0, (C) MAM:PAM 15:85, and (D) MAM:PAM 15:85 reagent quantities but not as core–shell particles.

After degradation there was an increase in *D*_h_ of both the MAM:PAM 100:0 and the PAM:MAM 100:0 nanogel compositions (Table 3), which will have likely led to a slight increase in the DCR values. There was a considerable increase in PDI of MAM:PAM 100:0 but not in PAM:MAM 100:0. The lack of change in the PDI of PAM:MAM 100:0 was attributed to the incomplete degradation of particles, due to the presence of permanent cross-links. This results in swelling (from 90 to 125 nm) but remaining particles contain enough permanent crosslinks to not noticeably increase the polydispersity. Due to the intensity-weighted measurement of particles by DLS the scattering from the relatively larger nanogels would mask any weaker scattering produced by the smaller soluble polymer chains and fragments of degraded nanogels. Indeed, the particle size distribution of PAM:MAM 100:0 particles was found to shift to larger diameters, while appearing monomodal (Fig. 4A). This is attributed to crosslinks breaking within the remaining nanogels, allowing the polymer chains to extend further into solution, increasing *D*_h_, but enough permanent crosslinks remain that the nanogels do not dissolve. The higher PDI of MAM:PAM 100:0 samples was attributed to the greatly reduced concentration of particles remaining, with residual particles swelling or dissolving, and with fewer of these particles needed to increase the PDI further. This was further supported by the appearance of a bimodal particle size distribution of MAM:PAM 100:0 particles after degradation, with particles seen at small (particle fragments) and larger (swollen partly degraded particles) diameter values (Fig. 4B). This is also shown in the number and volume distributions of the sample after degradation (ESI Fig. 4†), which indicate a much larger amount of smaller particles remaining compared to the intensity particle size distribution. PAM:MAM 100:0 particle size distributions in the same figure do not show a shift to smaller particle diameters. It was important to consider that differences in the conversion during the polymerisation of the nanogels might influence degradation, as a higher conversion might allow for more permanent cross-links to form. Therefore, ^1^H NMR was used to approximate residual monomer concentrations in both systems after synthesis to allow determination of the conversion. The conversion of PNIPAM was less than that of PNIPMAM (35% and 56%, respectively, Table 3). Unfortunately, it was not possible to quantify the BAC conversion for samples containing NIPAM due to overlaps NMR peaks with BAC as well as with polymer peaks. It is also worth noting that while nanogels are not soluble and therefore should not appear in the ^1^H NMR spectra, dangling chain ends may be soluble enough to be present. A full explanation of the determination of the conversion is given in the ESI.† The finding that MAM:PAM 100:0 had the highest conversion, yet highest degradation allowed us to rule out low conversion as a potential explanation for the differences between the degradation of the nanogels of only PNIPMAM (MAM:PAM 100:0) or only PNIPAM (PAM:MAM 100:0).

The core–shell nanogels, MAM:PAM 15:85 particles were also subjected to degradation conditions. These particles degraded considerably more than PAM:MAM 100:0 (5.6% residual DCR) but slightly less than MAM:PAM 100:0 particles. This was attributed to the increased PNIPAM content causing what was likely permanent cross-linking within the nanogels. While there no increase in the diameter of the particles after degradation, as seen with PAM:MAM 100:0, there was an increase in PDI, as seen with MAM:PAM 100:0. The PDI increase is attributed to the lower concentration of particles after degradation and a broader particle size distribution but unlike PNIPMAM particles was not bimodal (Fig. 4C). ESI Fig. 4† contains the number and volume distributions of the degraded nanogels also, which do not show the same shift to lower particle diameters seen with MAM:PAM 100:0 degraded samples. The lack of change in particle size after degradation was attributed to the increased permanent cross-linking of using PNIPAM in the nanogel synthesis, with some particles swelling due to the degradation of cross-links but not being able to dissolve fully. The particle size distributions were monomodal before and after degradation, so this increase was not an artefact of aggregates, as none were seen in the DLS cuvette. The conversion of NIPMAM was comparable in the core–shell system to that of MAM:PAM 100:0 after just 1 h (Table 3). At this point NIPAM monomer and other reagents were added to form the shell. 3 h after this addition, the conversion of NIPMAM had only increased by a further 14%, with NIPAM conversion being considerably lower than that of PAM:MAM 100:0 (19%, Table 3). These findings indicate that the outer shell of these particles was not just PNIPAM but a copolymer of the two monomers, with an estimated molar ratio of 89 : 11 PAM : MAM. The overall polymer molar ratio was 61 : 39 PAM : MAM. The composition of the shell is likely why the *T*_agg_ was the same as that of PAM:MAM 100:0. The core–shell structure and copolymer shell is also likely why swelling ratio values gradually decreased with increasing temperature in water rather than a sharp transition (Fig. 2). Polymers synthesised by Fundueanu *et al.* also show a variation in monomer ratio to final polymer composition, with monomer composition 67 : 33 having polymer composition 55 : 45 PAM : MAM and LCST of 35.9 °C.^47^ This data further confirms that presence of core–shell structure within the nanogels as the *T*_agg_ was considerably lower than the LCST of the similar composition polymer. Nanogels synthesised by Wiese *et al.* at monomer ratio 70 : 30 PAM : MAM also have a higher VPTT of 35 °C, which again supports core–shell structure on these nanogels (no conversion data is given in their publication).^48^

In order to further understand the effect of composition and structure of the nanogels on their degradation behaviour, samples were also synthesised using molar ratios of MAM : PAM 15 : 85 but as a copolymer rather than as core–shell particles. This was achieved by adding both monomers added as a mixed monomer feedstock at the start of the reaction but with all amounts kept the same as MAM:PAM 15:85 particles (see Table 3 for all analyses). The *T*_agg_ of these particles was determined to be 34.5 °C. This value was higher than those of both MAM:PAM 15:85 and PAM:MAM 100:0 (Table 2). This further supports the hypothesis that the sequential addition of the NIPAM after the NIPMAM formed a core–shell structure with predominately PNIPAM in the shell, as the presence of NIPMAM in the shell of the copolymer nanogel produced a higher *T*_agg_. Previous studies have shown minimal interpenetration of the shell polymer into the core when using these monomers, this is due to the core being hydrophobic at the reaction temperature and using a hydrophilic monomer.^52,53^ The particle size and residual DCR of these particles closely resemble those of PAM:MAM 100:0, rather than its core–shell counterpart (Table 3). With a zeta potential value before degradation of −28 mV, matching values of synthesised core–shell particles synthesised in this work. The residual DCR only decreased to 25% of the original value, indicating that more permanent cross-linking has taken place during polymerisation. It is unclear why the degradation was less than the core–shell system, but it may suggest the non-degradable cross-links predominately happen during the early stage of particle nucleation and growth. As during the polymerisation of the core–shell structure MAM:PAM 15:85 there was no NIPAM present during this stage. While for the copolymerisation approach MAM:PAM 15:85, PAM was present at the start of the polymerisation. This explanation is highly speculative and will require further work in the future to fully understand. For the copolymer MAM:PAM 15:85 the *D*_h_ values before and after degradation were similar to those of PAM:MAM 100:0 particles with the same trend of an increase in particles size but little change in the PDI. The particle size distribution can be seen in Fig. 4D. This data suggests that the nanogels were swelling rather than dissolving, with unimodal number and volume distributions (ESI Fig. 4†) showing no smaller particle diameters after degradation. Conversion data from ^1^H NMRs shows that high conversion values were not reached, when compared to MAM:PAM 15:85 core–shell conversion values, the PAM conversion was higher, with MAM conversion being lower. This was expected as PAM was reacted for 4 h instead of 3 h and MAM was now competing with increased PAM concentrations from the start of the reaction. Using the conversions by ^1^H NMR the ratio of PAM : MAM present in the nanogel was calculated to be 79 : 21. These results show the necessity of the core–shell composition of these particles. Furthermore, >94% degradation of the core–shell MAM:PAM 15:85 nanogels suggests that these particles will be suitable for use in future applications. As a result, the degradation of these particles in more physiologically relevant conditions was carried out.

### Degradation using physiologically relevant conditions

**Fig. 5:**
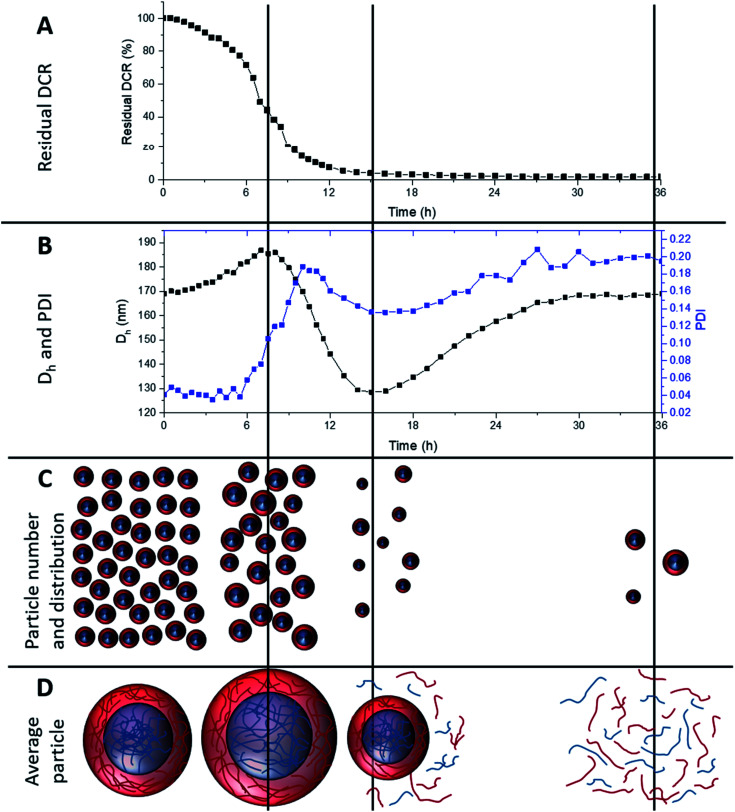
Overview of 15:85 MAM:PAM core–shell nanogel degradation over time at pH 7 and 10 mM DTT concentration. (A) Residual derived count rate over degradation time, (B) *D*_h_ and PDI of nanogel dispersion over degradation time, (C) representation of particle number and distribution over degradation time, and (D) average particle size or degradation product.

To investigate longer periods of degradation under physiologically relevant conditions degradations were carried out at pH 7, at 25 °C using 10 mM DTT concentration. This concentration was selected as 10 mM is the upper limit of intracellular reducing agent concentration.^32^ At this concentration, degradation occurs at a much slower rate. Fig. 5A shows the degradation of MAM:PAM 15:85 particles, as seen by a reduction in residual DCR with time. The DCR can be seen to decrease gradually until the DCR reached ∼2% of its original value over 36 h. This was consistent with the values achieved at 150 mM DTT concentration at pH 10, showing that the pH and DTT concentration has no obvious effect on the overall degradation of the particles besides the reduced rate. The initially slower reduction in the DCR can possibly be attributed to nanogel cross-links starting to be broken and as particles begin to dissolve the DCR reduction increases in rate. This eventually slowed and could be due to the lower concentration of particles as well as a reduction in the amount of DTT as it is consumed by both the disulfide reaction and the oxidation of DTT over time.^54^ From this data we can see that the particles can be degraded at physiologically relevant concentrations of reducing agent.

During the degradation using slower physiologically relevant degradation conditions it was also possible to monitor changes in *D*_h_ and PDI of the nanogels by DLS, shown in Fig. 5B. Initial and final *D*_h_ values for this degradation agree with those of the degradations completed at pH 10 and 150 mM DTT. This again supports that both degradation conditions used in this work result in similar particle degradations. Over the first 8 h the *D*_h_ values steadily increased, this can be attributed to cross-links within the nanogel being degraded and allowing the particles to swell. This is shown schematically in Fig. 5C and D along with a decrease in particle number. Subsequently the particle diameter can be seen to decrease, this was likely due to particles beginning to dissolve and become smaller, reaching a minimum value at around 15 h. This was again accompanied by a decrease in DCR (therefore likely the particle number) and likely leading to more polymer chains in solution (Fig. 5D). The *D*_h_ then increased again before levelling off at the original value. This coincides with the 36 h point where the DCR reached its lowest value. It can therefore be assumed that the smaller particles that can be fully degraded were doing so over this late period, becoming soluble polymer fragments. With the only remaining particles after 36 h being, on average, similar in diameter to the pre-degradation particle diameter. As seen in the pH 10 degradations, using 150 mM DTT concentration, the PDI increases from 0.04 to around 0.2, however the PDI can also be seen to fluctuate over the course of the degradation. The fluctuation in PDI follows a similar pattern to that of the variation in *D*_h_, except after 6 h an initial more pronounced increase in PDI for the first ∼10 h. This was attributed to the particles swelling at different rates depending on the amount of cross-linker they contained. The decrease in PDI however does not start for another few hours after the diameter has started to decrease. This was attributed to particles beginning to dissolve, decreasing the diameter, but this is likely to also initially increase the PDI as particles dissolve at different rates. Eventually the PDI decreased as all the particles that will dissolve begin to do so, before increasing again to the final value. The increased final value is attributed to particles that cannot fully degrade, resulting in a broad particle size distribution (PSD), PSD graphs before and after degradation can be seen in ESI Fig. 3.† This analysis shows that the MAM:PAM 15:85 core–shell nanogels predominately degrade into soluble polymer fragments with a very low amount of the nanogels remaining in the swollen form. Unfortunately, the authors could not obtain molecular weights of the degradation product of MAM:PAM 15:85 by size exclusion chromatography. This was due to fragments of the nanogels blocking 0.2 μm PTFE filters which prevented complete sample analysis.

## Conclusions

Degradable core–shell nanogels of varying compositions of PNIPAM and PNIPMAM were synthesised. The *T*_agg_ of the particles was able to be tuned depending on the monomer ratios and the chemistry of the shell polymer. It was determined that to synthesise nanogels that aggregate below 37 °C a monomer content split of a 15% NIPMAM core and 85% NIPAM shell was needed, MAM:PAM 15:85. Degradation of these particles as well as reference systems consisting of only PNIPAM and PNIPMAM was attempted (PAM:MAM 100:0 and MAM:PAM 100:0, respectively). At pH 10 and 150 mM reducing agent concentration PNIPAM particles were only ∼73% degradable, whereas those composed entirely of PNIPMAM were almost completely degradable ∼97.5%. This is consistent with previous reports of PNIPAM based systems that generate permanent cross-links. The degradability of MAM:PAM 15:85 was similar to that of MAM:PAM 100:0 nanogels at ∼94%. Synthesis of particles of an 15 : 85 monomer ratio as a one-pot system were shown to be not as degradable and have a higher *T*_agg_, therefore indicating a core–shell structure of MAM:PAM 15:85. As a result the degradability was also investigated at physiologically relevant pH and reducing agent concentration, pH 7 and 10 mM. While this increased the degradation time to 36 h, the total inferred amount of degradation was the same. The high level of degradation shown in this work combined with the ability to tune the thermoresponsive behaviour of the nanogels will be useful in the applications of nanogels in biological applications. Where the degradation offers the potential to prevent persistent accumulations of the nanogels and the tuneable aggregation behaviour can be utilised in *in situ* forming systems and drug delivery platforms.

## Author contributions

Dominic Gray, conceptualization, investigation, resources, writing – original draft preparation, visualization. Adam Town, conceptualization, visualization, investigation. Edyta Niezabitowska, investigation, writing – review & editing. Steve Rannard, writing – review & editing, supervision. Tom McDonald supervision, project administration, visualization, conceptualization, writing – review & editing, funding acquisition.

## Conflicts of interest

There are no conflicts to declare.

## Supplementary Material

RA-012-D1RA07093B-s001
