# Supplementary material for: Dual-responsive degradable core–shell nanogels with tuneable aggregation behaviour
Source: RSC Adv. 2022 Jan 13;12(4):2196–206. doi: 10.1039/d1ra07093b (PMC8979186; doi:10.1039/d1ra07093b)
Supplement: RA-012-D1RA07093B-s001 [file RA-012-D1RA07093B-s001.pdf]

Electronic Supplementary Material (ESI) for RSC Advances.
This journal is © The Royal Society of Chemistry 2021

Dual-responsive degradable core-shell nanogels with tuneable aggregation behaviour

Supplementary Information

Supplementary Table 1. Table of results comparing MAM:PAM 100:0 nanogels synthesised at 160 mL and 64 mL total water volume scales.

Sample	Hydrodynamic Diameter[a] (nm)	PDI	Swelling Ratio[b]	T_{agg} (°C)	Residual DCR (%)
160 mL	156	0.02	1.31	43	0.9
64 mL	143	0.02	1.35	43	2.5

[a] Hydrodynamic diameter of a 1 mg ml^{-1} aqueous dispersion at 25 °C from DLS data, value is the mean value of triplicate measurements. [b] Swelling ratio calculated using D_h (Hydrodynamic diameter). D_h (15 °C)/D_h (55 °C).

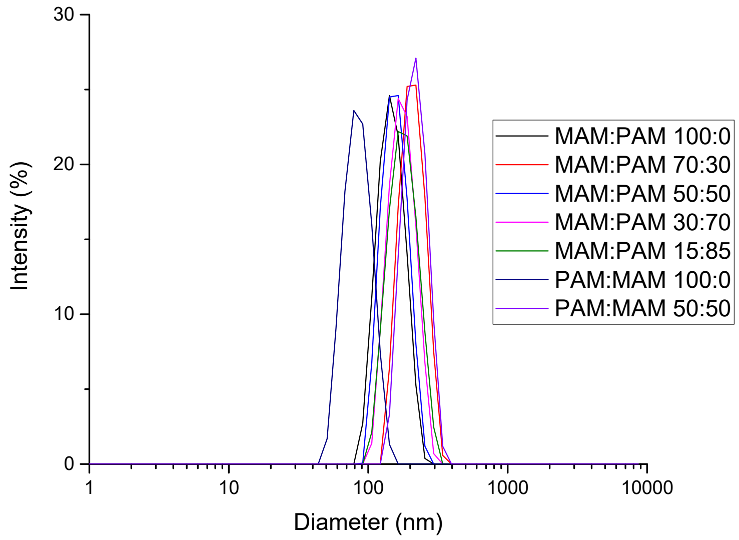

Supplementary Figure 1. Particle size distributions by intensity of core-shell nanogels from DLS data obtained at 25 °C and 1 mg mL^{-1} concentration.

To make a direct comparison of the effect of pH conditions, two samples of 1 mg mL^{-1} dispersions of MAM:PAM 50:50 were adjusted to pH 7 and at 150 mM DTT concentration. One sample was then adjusted to pH 10 with photographs taken before addition and 2 seconds after addition with shaking. The change in turbidity was considerable for the sample at pH 10 but pH 7 showed no change in that timeframe. This can be seen visually in Supplementary Figure 2.

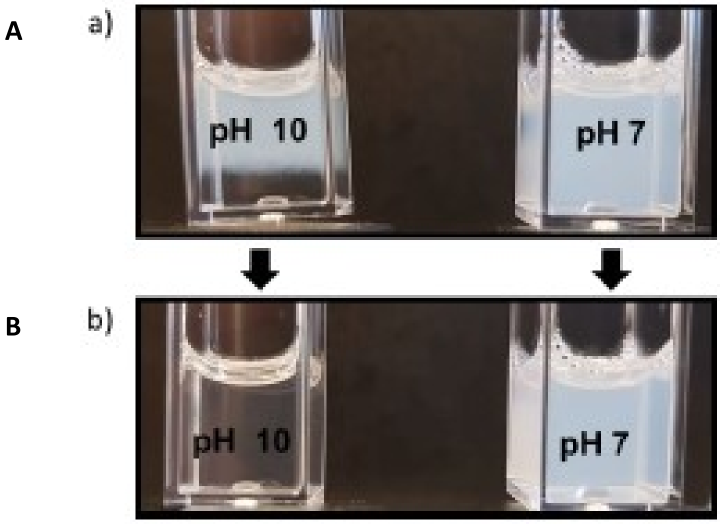

Supplementary Figure 2. Comparison photographs of the change in turbidity of 1 mg mL^{-1} dispersions of MAM:PAM 50:50 at 150 mM DTT concentration A - as the pH was adjusted for the pH 10 sample (left), B - 2 seconds after addition with manual shaking.

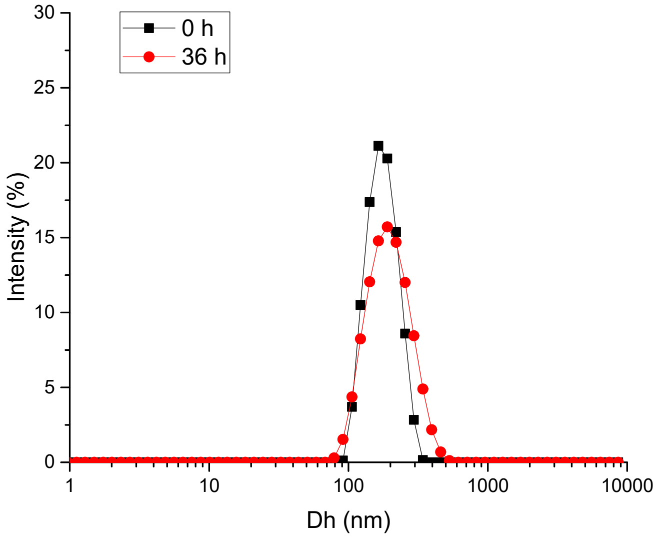

Supplementary Figure 3. Particle size distributions by intensity of MAM:PAM 15:85 core-shell nanogels from DLS data obtained at 25 °C and 1 mg mL^{-1} concentration. Before (0 h) and after (36 h) degradation at pH 7 and 10 mM DTT concentration.

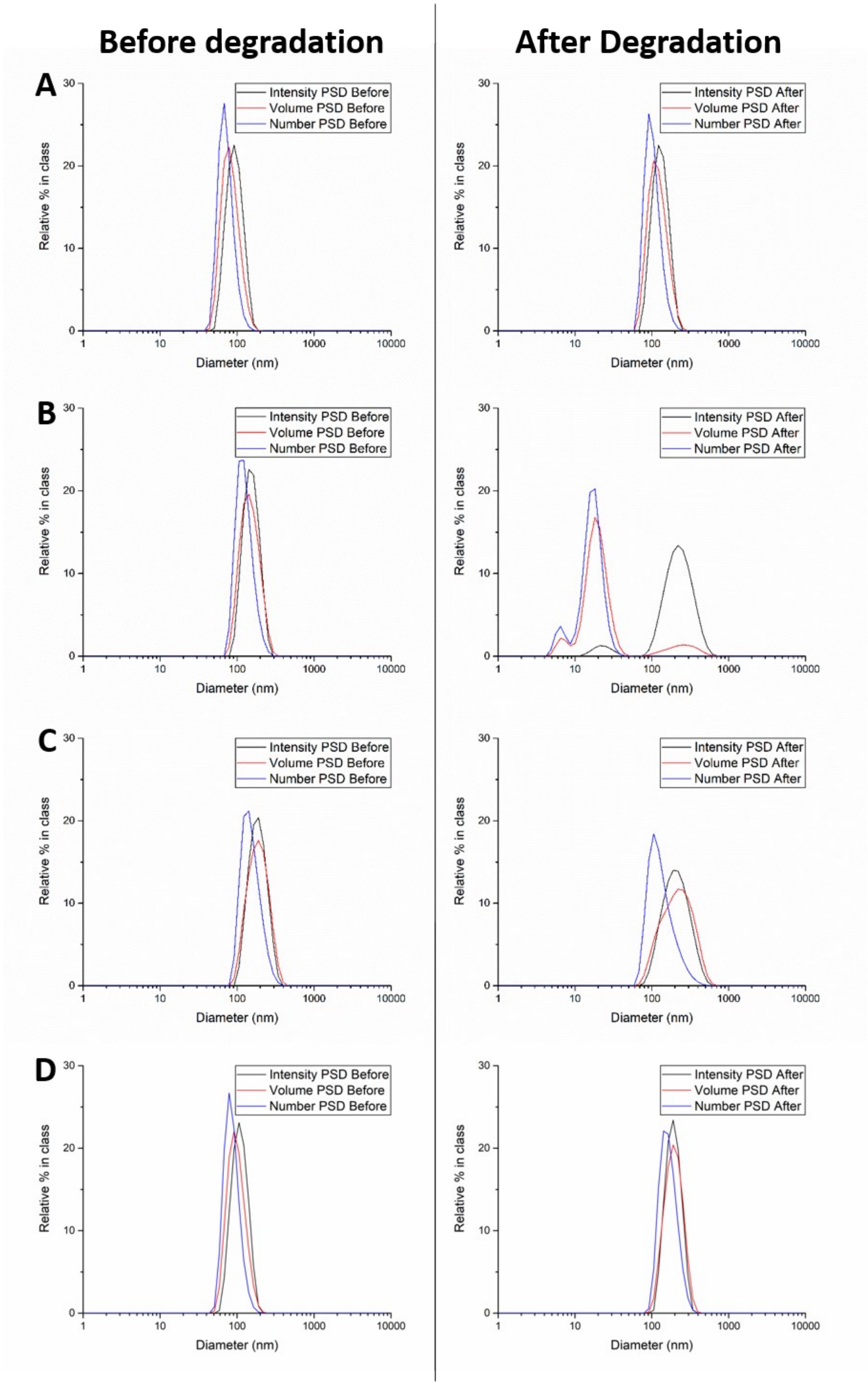

Supplementary Figure 4. Comparisons of intensity, volume and number particle size distributions by DLS of the following samples: A. PAM:MAM 100:0, B. MAM:PAM 100:0, C. MAM:PAM 15:85, D. MAM:PAM 15:85 reagent ratio not core shell. Graphs in the left column are before degradation and graphs in the right column are after degradation.

Calculation of ^{1}H NMR conversion

NIPMAM conversion is calculated from ^{1}H NMR spectra obtained at reaction times t = 0 h, 1h (before and after shell addition) and 4 h (final timepoint, reaction complete) for MAM:PAM 15:85. For MAM:PAM 100:0 and MAM:PAM 15:85 not core-shell particles samples taken at t = 0 h and t = 4 h only were used. Peaks at 5.47-5.67 ppm and 5.3-5.47 ppm (see Supplementary Figure 4 peaks (a) and (b), respectively) were integrated and the average value of the two at 4 h was divided by the value at 0 h and multiplied by 100. NIPMAM conversion was calculated assuming that no conversion takes place immediately on the addition of aqueous solutions of BAC with NIPAM and of KPS, as the dilution of the reaction mixture changes the ratios of reagents.

NIPAM conversion was calculated from ^{1}H NMR spectra from samples taken at timepoints 0 h and 4 h (final) for PAM:MAM 100:0 and MAM:PAM 15:85 not core-shell particles syntheses. For MAM:PAM 15:85 core-shell particles samples taken at t = 1 h (after shell addition) and t = 4 h (final) were used. Integreation of a peak at 6.1-6.143 ppm (see Supplementary Figure 4 peak (c)) was used to calculate monomer conversion as all other NIPAM related peaks overlap with other reagents.

For BAC conversions by ^{1}H NMR integration of a peak at 5.72-5.85 ppm was used. This could only be calculated accurately when NIPAM was not added to the polymerization: MAM:PAM 100:0 and timepoints at 0 h and 1 h (before shell addition) of MAM:PAM 15:85 core-shell. In MAM:PAM 15:85 4 h this overlaps signal overlaps with NIPAM

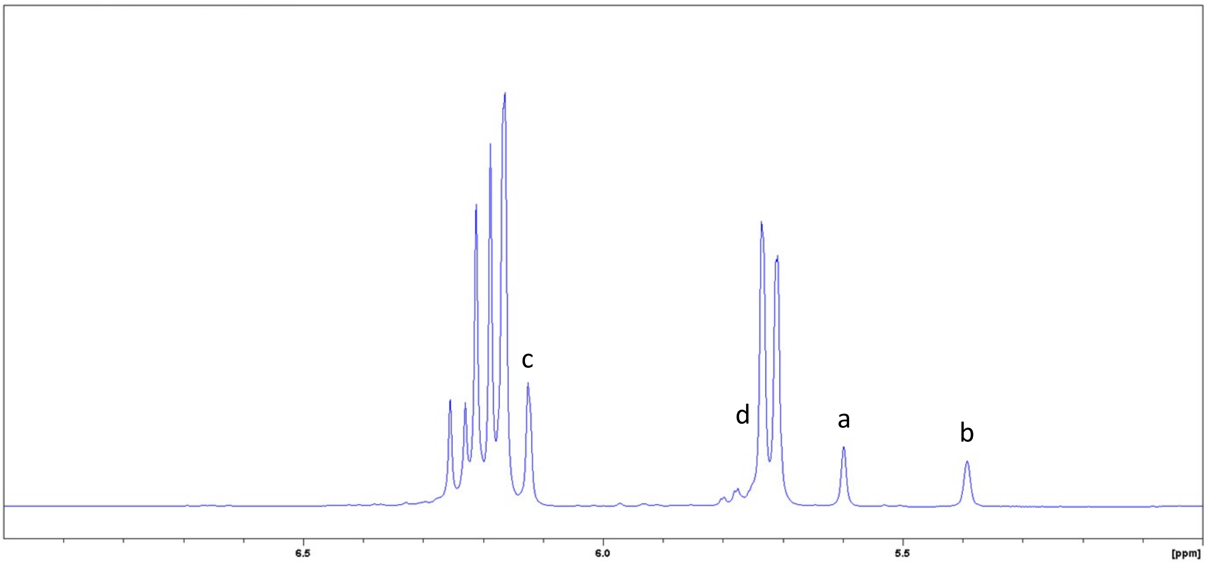

Supplementary Figure 4. ^{1}H NMR of MAM:PAM 15:85 at a reaction time of 4 hours, showing regions of interest to this study between 5 and 7 ppm.

resonances and so cannot be used to calculate conversion (d).

MAM:PAM 15:85 polymer ratio calculation

The following calculation is for to determine the polymer ratio of PNIPAM to PNIPMAM in the shell of the nanogel:

Outer layer conversions are 14 % MAM and 19 % PAM by ^{1}H NMR

Total amount of NIPMAM in the shell synthesis = 0.265 g, 14 % conversion = 37.1 mg

Moles of PNIPMAM (shell) = 0.29 mmoles

Total amount of NIPAM in the shell synthesis = 1.336 g, 19 % conversion = 253.83 mg

Moles of PNIPAM (shell) = 2.24 mmoles

Polymer ratio in the shell MAM:PAM 11:89

Total amount of NIPMAM in the core synthesis = 0.265 g, 55 % conversion = 145.8 mg

Moles of PNIPMAM (core) = 1.15 mmoles

Total molar ratio (core+shell) MAM:PAM = 39:61

MAM:PAM 15:85 not core-shell particles polymer ratio

Calculated from monomer conversion from ^{1}H NMR:

Moles of PNIPMAM at 46 % conversion = 0.1219 g = 0.96 mmoles

Moles of PNIPAM at 30 % conversion = 0.4008 g = 3.54 mmoles

Total moles of polymer = 4.5 mmoles

Ratio = MAM:PAM 21:79
